# The Roles of Mast Cells in Parasitic Protozoan Infections

**DOI:** 10.3389/fimmu.2017.00363

**Published:** 2017-04-06

**Authors:** Fangli Lu, Shiguang Huang

**Affiliations:** ^1^Department of Parasitology, Zhongshan School of Medicine, Sun Yat-sen University, Guangzhou, China; ^2^Key Laboratory of Tropical Disease Control Sun Yat-sen University, Ministry of Education, Guangzhou, China; ^3^School of Stomatology, Jinan University, Guangzhou, China

**Keywords:** mast cell, *Plasmodium* spp., *Leishmania* spp., *Trypanosoma* spp., *Toxoplasma gondii*

## Abstract

Protozoan parasites such as *Plasmodium* spp., *Leishmania* spp., *Trypanosoma* spp., and *Toxoplasma gondii* are major causes of parasitic diseases in both humans and animals. The immune system plays a critical role against protozoa, but their immune mechanism remains poorly understood. This highlights the need to investigate the function of immune cells involved in the process of parasite infections and the responses of host immune system to parasite infections. Mast cells (MCs) are known to be central players in allergy and anaphylaxis, and it has been demonstrated that MCs have crucial roles in host defense against a number of different pathogens, including parasites. To date, there are many studies that have examined the interaction of helminth-derived antigens and MCs. As one of the major effector cells, MCs also play an important role in the immune response against some parasitic protozoa, but their role in protozoan infections is, however, less well characterized. Herein, we review the current knowledge about the roles of MCs and their mediators during infections involving highly pathogenic protozoa including *Plasmodium* spp., *Leishmania* spp., *Trypanosoma* spp., and *T. gondii*. We offer a general review of the data from patients and experimental animal models infected with the aforementioned protozoa, which correlate MCs and MC-derived mediators with exacerbated inflammation and disease progression as well as protection against the parasitic infections in different circumstances. This review updates our current understanding of the roles of MCs during parasitic protozoan infections, and the participation of MCs in parasitic protozoan infections could be of a potential therapeutic target.

## Introduction

*Plasmodium* spp., *Leishmania* spp., *Trypanosoma* spp., and *Toxoplasma gondii* are some of the most important medical protozoan parasites that cause diseases in humans. *Plasmodium* spp. is a group of mosquito-borne parasitic protozoa. After being bitten by an *Anopheles* mosquito, sporozoites penetrate the liver cells of the host and produce thousands of free merozoites, which invade erythrocytes and then burst the cells to release the merozoites to invade other erythrocytes and cause clinical symptoms (1). *Leishmania* spp. comprises several species and causes leishmaniasis, which affects more than 300 million people worldwide (2). This parasite has a complex life cycle composed of two distinct stages: the promastigote form found in the female sandfly vector and the amastigote form replicated in the mammalian host (3). *Trypanosoma brucei* causes the fatal illness human African trypanosomiasis (4), which is adapted to parasitize the mammalian bloodstream after inoculation by the tsetse fly (*Glossina* spp.). *Trypanosoma cruzi* causes American trypanosomiasis or Chagas disease. This parasite chronically infects millions of people, and up to 30% of the infected individuals ultimately develop chronic cardiomyopathy or gastrointestinal disease. Transmission of this parasite occurs when trypomastigotes in vector (triatomine bug) feces enter bite wounds, mucous membranes of the nose, oral cavity, or conjunctiva of the new host. In addition, transmission can also occur through an oral route by ingestion of food contaminated with triatomine bugs or their feces (5, 6). *T. gondii* is spreading all over the world, which can infect a vast number of intermediate hosts and causes toxoplasmosis in both humans and animals (7). Toxoplasmic encephalitis is a subsequent risk for all severely immunocompromised patients (8). Moreover, infection during pregnancy may cause serious lesions to the fetus through congenital infection (9).

Mast cells (MCs) are tissue-resident, granule-containing cells, which participate in the regulation of innate and adaptive immune responses (10). In healthy individuals, MCs are involved in tissue homeostasis, tissue repair, and host defense *via* the release of different kind of pro-inflammatory mediators, proteases, and cytokines (11). Degranulation of MCs is essential for host defense against parasitic infections (12). It is well known that MCs play an important role in parasitic helminth infections (13). Accumulating evidences have demonstrated that MCs have pivotal roles in parasitic protozoan diseases (14, 15); this has led us to focus on the role of MCs in the immune responses against parasitic infections including *Plasmodium* spp., *Leishmania* spp., *Trypanosoma* spp., and *T. gondii*. The main function of MCs in the aforementioned protozoa is summarized in Table 1.

**Table 1:** **The function of mast cells (MCs) in *Plasmodium* spp., *Leishmania* spp., *Trypanosoma* spp., and *Toxoplasma gondii* infections**.

Parasite	MC/disease	Animal model/patient sample	Mechanism	No involvement	Reference
*Plasmodium* spp.	In the skin of patients or animals with *Plasmodium* infection	Patients with *Plasmodium falciparum* malaria	Significantly increased MC degranulation was correlated with parasitemia and disease severity		Wilainam et al. (16)
		Swiss mice exposed to *Anopheles gambiae* mosquitoes infected with *Plasmodium berghei* NK65	MCs were observed in the vicinity of sporozoites at 1 h after mosquito bite		Choumet et al. (17)
	In the brains of mice with *Plasmodium* infection	C57BL/6-WBB6F_1_-*W/W^v^* (*W/W^v^*) and wild-type WBB6F_1_^+/+^ (+/+) mice infected with *P. berghei* ANKA	+/+ mice had lower parasitemia and mortality, with higher tumor necrosis factor levels compared to *W/W^v^* mice		Furuta et al. (18)
		MC-deficient and basophil-depleted C57BL/6 mice infected with *P. berghei* ANKA		MCs and basophils were not involved in the development of experimental cerebral malaria	Porcherie et al. (19)
		H3R^−/−^ mice infected with *P. berghei* ANKA	The severity of cerebral malaria was correlated with the increased plasmatic levels of histamine in H3R^−/−^ mice		Beghdadi et al. (20)
		Histidine decarboxylase-deficient (HDC^−/−^) C57BL/6 mice infected with *P. berghei*	HDC^−/−^ mice were highly resistant to *P. berghei* infection, with a drastic reduction of brain-infiltrating T cells and decreased expression of adhesion molecules		Beghdadi et al. (21)
			Infected wild-type C57BL/6 mice showed prolonged survival after treatment with antihistamine drugs		
	In the intestines of animals with *Plasmodium* infection	Mice infected with *Plasmodium yoelii*	Increased ileal mucosal MCs was positively correlated with elevated parasitemia and ileal interleukin (IL)-4 levels		Chau et al. (22)
		Rhesus macaques infected with *Plasmodium fragile*	Ileal mastocytosis and increased plasma histamine levels were exhibited		Potts et al. (23)
		Antihistamine treatment of *P. yoelii*-infected CBA/J mice	MCs and histamine were involved in increased intestinal permeability during *Plasmodium* infection		
*Leishmania* spp.	In the skin of patients and animals with *Leishmania* infection	Patients with cutaneous leishmaniasis caused by *Leishmania braziliensis*	There was a positive association between the disease duration and MCs count in the skin biopsy		Tuon et al. (24)
		Susceptible (BALB/c) and resistant (C57BL/6 and CBA/T6T6) mice infected with *Leishmania major*	MC numbers were significantly increased in the upper dermis of BALB/c but not in those of C57BL/6 and CBA/T6T6 mice after *L. major* infection		Saha et al. (25)
		MC-deficient *Kit^W-sh^*/*Kit^W-sh^* mice infected with *L. major*	Significantly enhanced lesion progression and lesional parasite burdens were observed, accompanied by significantly decreased levels of IFN-γ and IL-17A		Dudeck et al. (26)
		Dogs infected with *Leishmania infantum/chagasi*	Dermic inflammatory reaction with many degranulated MCs was observed		Calabrese et al. (27)
		Skin samples from dogs naturally infected with *L. infantum*	Increased number of MCs in the skin was correlated with clinical progression of canine visceral leishmaniasis		Menezes-Souza et al. (28)
		MC-deficient *Kit^W^/Kit^W-v^* mice and normal *Kit*^+^*^/^*^+^ mice infected with *L. major*	Increased lesion sizes and lesional parasitic loads and reduced locally infiltrating cells were observed in *Kit^W^/Kit^W-v^* mice		Maurer et al. (29)
		C57BL/6 or BALB/c mice infected with *L. major*		MCs had no role on lesion size development, parasitic load, and immune cell phenotypes during murine cutaneous leishmaniasis	Paul et al. (30)
	In visceral leishmaniasis	Dogs naturally infected with *L. infantum*	Lower number of MCs was observed in the lamina propria of gastrointestinal tract of the infected dogs		Pinto et al. (31)
	In ocular leishmaniasis	C57BL/10 and BALB/c mice, both susceptible to leishmaniasis, infected with *Leishmania amazonensis* by intravitreal injection and instillation, respectively	Many intact MCs were presented in the conjunctiva of both strains of mice at 30 days after infection, while degranulated MCs were observed in the conjunctiva of BALB/c mice at 60 days after infection		Calabrese et al. (32)
*Trypanosoma* spp.	In chagasic megacolon	Patients with Chagas disease caused by *Trypanosoma cruzi*	The number of tryptase-positive MCs was significantly increased in the lamina propria, muscle layer, or myenteric plexus region		Martins et al. (33)
			A greater MC count and more fibrosis were found in the colon musculature with megacolon compared to that without megacolon		Pinheiro et al. (34)
			Patients with megaesophagus had increased numbers of tryptase-positive MCs		Martins et al. (14)
		Swiss mice infected with *T. cruzi* Y strain	Significantly increased number of MCs was observed in the muscular layer of mice with chagasic megacolon		Campos et al. (35)
	In Chagas heart disease	CBA mice infected with *T. cruzi* plus cromolyn treatment	Greater parasitemia, higher mortality, myocarditis, and cardiac damage were found in the infected mice treated with MC stabilizer		Meuser-Batista et al. (36)
		Patients with chronic Chagas disease	MC chymase density was associated with the intensity of myocardium fibrosis of chronic Chagas disease		Roldão et al. (37)
	In *Trypanosoma brucei* infection	Rats infected with *T. brucei*		The levels of MCs in the intestines of *T. brucei*-infected rats were similar to those of uninfected controls	Gould and Castro (38)
*T. gondii*	In toxoplasmic encephalitis	A patient with meningoencephalitic toxoplasmosis	Systemic cutaneous and gastrointestinal mastocytosis were observed		Koeppel et al. (39)
	In ocular *T. gondii* infection	*Calomys callosus* inoculated with *T. gondii* RH strain *via* the conjunctiva	Significantly increased MC number and MC activation were observed in the ocular tissues		Gil et al. (40)
	In oral *T. gondii* infection	MC-deficient mice (W/W^v^*)* and control +/+ orally infected with cysts of *T. gondii* ME49 strain	Rapid lethality of *T. gondii* infection and decreased serum IFN-γ levels were observed in the infected mice in the absence of MCs		Cruz et al. (41)
	In intraperitoneal *T. gondii* infection	*C. callosus* infected with *T. gondii* RH strain	The number of degranulated MCs was significantly higher than that of intact MCs, with a remarkable increase in the influx of neutrophils and Mφ toward the peritoneal cavity		Ferreira et al. (42)
		MC-deficient *Kit^W^*/*Kit^W-v^* mice infected with *T. gondii* RH strain	The influx of Ly6G^+^ cells toward the peritoneal cavity was significantly reduced compared to control littermates		Del Rio et al. (43)
		Kunming outbred mice infected with *T. gondii* RH strain	Significantly increased parasite burden, tissue inflammation, and Th1 cytokine mRNA levels were detected in the livers and spleens of infected mice treated with an activator of MC release		Huang et al. (44)
			Significantly decreased parasite burden and tissue inflammation, and significantly increased Th2 cytokine mRNA levels were detected in the livers and spleens of infected mice treated with an inhibitor of MC release		

## MCs in *Plasmodium* spp. Infection

### MCs in the Skin of Patients and Animals with *Plasmodium* Infection

Mast cells are abundant in tissues exposed to the external environment, including the skin (45). Malaria parasites may promote malaria pathogenesis by triggering MCs. *Plasmodium berghei*-infected *Anopheles gambiae* mosquito saliva can trigger mouse dermal MC degranulation as little as 5 min after the mosquito bite. One hour after the bite, MCs were observed in the vicinity of sporozoites on skin sections from mice bitten by *P. berghei*-infected *An. gambiae* mosquitoes (17). Saliva-induced activation of dermal MCs causes lymph node swelling *via* the recruitment of T cells, B cells, dendritic cells (DC), and monocytes/macrophages (Mφ) as well as neutrophils (46, 47). Importantly, it was shown that there was an increase in MC activation and degranulation in the skin dermis of severe *Plasmodium falciparum* malaria patients, compared to controls. The percentage of MC degranulation was significantly correlated with parasitemia and disease severity, which are relevant to MC mediators (16).

### MCs in Cerebral Malaria (CM)

Mast cells are found in the central nervous system, especially along the blood vessels and leptomeninges (48). Kenyan children with mild and severe malaria were shown to have increased plasma levels of Flt3 ligand (Flt3L) (15). Elevated serum Flt3L levels and DC expansion were found in patients infected by *P. falciparum* and mice with *Plasmodium* infection. MCs are an important source of Flt3L, a soluble cytokine that influences DC function, with the subsequent activation of pathogenic CD8^+^ T cells, a critical effector of the disease (49). After infection with *P. berghei* ANKA, compared to MC-deficient WBB6F_1_-*W/W^v^* mice, the control littermate WBB6F_1_^+/+^ C57BL/6 mice had lower parasitemia and mortality with higher tumor necrosis factor (TNF) levels. Malarial antigens from *P. berghei* ANKA are able to stimulate Mφ and MCs to secrete TNF *in vitro*. An *in vivo* study further demonstrated that MCs are a critical source of TNF in addition to Mφ and T cells in murine malaria. Therefore, MCs and MC-derived TNF play an important role in protection against experimental cerebral malaria (ECM) (18). Furthermore, *P. berghei* ANKA peroxiredoxin induces a significant amount of MC-derived TNF secretion from IgE-mediated protection through FcεRI on MCs and innate immunity by means of toll-like receptor (TLR) 4 with myeloid differentiation primary response gene 88 and MD-2 and plays a role in innate and acquired immune responses in malaria (50). Conversely, one study reported that malaria developed in MC-deficient and basophil-depleted C57BL/6 mice infected with *P. berghei* ANKA was similar to that developed in wild-type mice, suggesting that MCs and basophils were not involved in malaria pathogenesis in this model (19).

Histamine has four different receptors, namely H1R, H2R, H3R, and H4R, which mediate numerous different effects (51). Histamine is the major MC mediator in malaria, and its signaling has been associated with the severity of *P. falciparum* malaria (21). In addition, the significant elevation of the blood concentrations of IgE and IgE-antimalarial antibodies has also been linked to the disease severity in *falciparum* malaria patients (52). As a DC modulator, especially during early phases of the immune response, histamine causes increased vascular permeability and subsequent extensive vascular damage to endothelial cells during malaria infection (47). Experiments have demonstrated that histamine binding to H1R and H2R increases the susceptibility of infection with *P. berghei* in H1R^−/−^ and H2R^−/−^ mice (21). H3R^−/−^ mice infected with *P. berghei* ANKA have an accelerated onset of CM and mortality, accompanied by an earlier loss of blood–brain barrier integrity, earlier formation of hemorrhagic lesions, higher sequestration of CD4^+^ and CD8^+^ T cells in the brain, and higher serum histamine levels compared to C57BL/6 wild-type mice. The severity of CM is related to the increased plasmatic levels of histamine in H3R^−/−^ mice during the infection (20). Mice genetically deficient in the histidine decarboxylase (HDC^−/−^) gene, thus lacking histamine, were highly resistant to lethal infection by *P. berghei* ANKA and *P. berghei* NK65, associated with decreased brain-infiltrating T cells and expression of adhesion molecules. After treatment with antihistamine drugs, mice infected with *P. berghei* had prolonged survival compared to infected mice without antihistamine treatment (21).

Vascular endothelial growth factor (VEGF) is both neuroprotective and pro-inflammatory in the brain. VEGF was shown to accumulate intracellularly in *P. falciparum*-infected red blood cells *in vitro*, and inhibition of VEGF receptor (VEGFR)-2 signaling reduced intraerythrocytic growth of *P. falciparum* (53). VEGF and soluble VEGFR (sVEGFR)-2 are increased in CM patients compared to healthy adults (54). Plasma VEGF concentrations in Kenyan children with CM are associated with an increased risk of neurological sequelae (55). sVEGFR-1 may play a pathological role during chronic placenta malaria and hypertension in first-time mothers (56). Both *P. falciparum* and *Plasmodium vivax* crude antigens induce VEGF release from the human MC line HMC-1 or the human basophilic cell line KU812 *in vitro*. Increased parasitemia of *P. berghei* ANKA was observed in anti-VEGF Ab-treated mice compared to non-treated mice (54). Furthermore, VEGF was shown to promote malaria-associated acute lung injury induced by *P. berghei* ANKA in mice (57). The pro-inflammatory cytokine interleukin (IL)-33 is strongly enhanced in infants (<5 years) with severe malaria from *P. falciparum* infection (58). IL-33 contributes to the stimulation and release of VEGF in human MCs (hMCs) (59). Conversely, IL-33 prevents the development of ECM in C57BL/6 mice infected with *P. berghei* ANKA and reduces the production of inflammatory mediators IFN-γ, IL-12, and TNF-α (60).

### MCs in the Intestines of Animals with *Plasmodium* Infection

In the gastrointestinal tract, MCs regulate vascular and epithelial permeability, ion secretion, angiogenesis, peristalsis, fibrosis, tissue repair, and innate and adaptive immunity (61). Increased numbers of mucosal MCs (MMCs) in the ileal villi and crypts and increased histamine levels in the ileum were detected in *Plasmodium yoelii*-infected mice. The increase in ileal MMCs was positively correlated with elevated parasitemia and IL-4 mRNA levels in the same tissue. An additional study found that *P. yoelii nigeriensis*-infected mice develop an l-arginine deficiency, which is associated with intestinal mastocytosis, elevated levels of plasma histamine, and enhanced intestinal permeability (22). *Plasmodium fragile*-infected rhesus macaques have been shown to exhibit ileal mastocytosis and increased plasma histamine levels. Antihistamine treatment during *P. yoelii* infection results in decreased intestinal permeability in CBA/J mice, suggesting that MCs and histamine are involved in increased intestinal permeability during *Plasmodium* infection (23).

## MCs in *Leishmania* spp. Infection

Leishmaniasis is caused by several different species of *Leishmania*, and each infection has a different clinical outcome; human leishmaniasis is usually classified as cutaneous, mucocutaneous, or visceral. *Leishmania* parasites primarily live in Mφ (25), but *Leishmania major* and *Leishmania infantum* promastigotes can also bind to MC membranes and infect MCs (62).

### MCs in the Skin of Patients and Animals with *Leishmania* Infection

Cutaneous leishmaniasis is an important public health concern in many parts of the world, especially in Africa (63). MCs degranulate and release inflammatory mediators such as TNF-α after infection and recruit polymorphonuclear leukocytes (PMNs) to the site of infection (64). MCs can be important in cutaneous leishmaniasis and are involved in healing lesions. The MC count was higher in the skin biopsy of patients with cutaneous leishmaniasis caused by *Leishmania braziliensis* with earlier healing after treatment, and there was a positive association between the disease duration and MC count (24).

After infection of *Leishmania amazonensis* in susceptible (C57BL/10 and CBA), relatively resistant (DBA/2), and resistant (C3H.He) mice, the primary lesions in footpads and draining lymph nodes showed a predominance of eosinophils and MCs in the initial phase of infection in all the infected mice (65). MC numbers increased significantly in the upper dermis in susceptible (BALB/c) but not in resistant (C57BL/6 and CBA/T6T6) mice after *L. major* infection. However, the number of degranulating MCs was higher in CBA/T6T6 mice during early *L. major* infection. In addition, MC-derived cytokines, such as TNF-α, play a proparasitic role in a susceptible strain (BALB/c) of mice, but an antiparasitic role in resistant strains (C57BL/6 and CBA/T6T6) of mice, suggesting that the susceptible and resistant mouse strains may have different modes of producing the antileishmanial immune response by differential regulation of MC function (25). After *L. major* inoculation, MC activation and parasite uptake by skin-resident Mφ occurred, followed by neutrophil and monocyte immigration and DC activation. Therefore, MC-dependent recruitment of Mφ, PMN, and DC to the skin is involved in controlling leishmaniasis (66). Furthermore, using MC-deficient *Kit^W-sh^*/*Kit^W-sh^* mice for infection with *L. major* promastigotes results in a worse disease outcome, e.g., significantly enhanced lesion progression and lesional parasite burdens, accompanied by significantly decreased levels of IFN-γ and IL-17A, but significantly increased IL-4 and IL-10, compared to wild-type mice, indicating that MCs play a crucial role against *Leishmania* parasites by promoting Th1 and Th17 responses *in vivo* (26). *L. infantum*/*chagasi*-infected skins, from dogs of two different leishmaniasis endemic areas of Brazil, showed different skin infection patterns; however, dogs from both areas showed dermic inflammatory infiltrates composed of numerous degranulated MCs compared to normal skin, indicating that MCs modulate the immune response and participate in the host defense against *Leishmania* infection (27). Dogs naturally infected with *L. infantum* showed increased inflammatory infiltrates in the skin of animals with severe forms of canine visceral leishmaniasis and a high parasite density. The increased number of Mφ and decreased number of lymphocytes, eosinophils, and MCs in the skin correlate with clinical progression of canine visceral leishmaniasis (28).

After susceptible BALB/c and resistant C57BL/6 mice were infected with *L. major* and pretreated with compound 48/80 (a MC activator), both BALB/c and C57BL/6 mice displayed smaller lesions in footpads compared to controls, indicating that MC degranulation contributes to susceptibility to *L. major* infection, and in the absence of granulated MCs, BALB/c and C57BL/6 mice had increased resistance to *L*. *major* infection. Although IL-4 and MC degranulation are important to *Leishmania*-associated pathogenesis, the MC-mediated susceptibility seems to be independent of IL-4 (67). MC-deficient C57BL/6-*Kit^W^/Kit^W-v^* mice and congenic wild-type *Kit*^+^*^/^*^+^ mice were infected with metacyclic promastigotes of *L. major* by intradermal injections, resulted in significantly increased lesion sizes and lesional parasitic loads, and significantly reduced locally infiltrating cells in *Kit^W^/Kit^W-v^* mice. In addition, pronounced MC degranulation was observed in infected skin sites after intradermal injection of *L. major* in C57BL/6 mice, indicating that MCs provide protection against *L. majo*r infection (29). An *in vitro* study showed that stimulation with *Leishmania mexicana* lipophosphoglycan led to a significant increase in degranulation in bone marrow-derived MCs (BMMCs) from BALB/c mice compared to BMMCs from C57BL/6 mice. Moreover, an *in vivo* study showed that after infection with *L. mexicana*, the number of MCs increased more rapidly and to a greater level, with significantly higher levels of parasites in the lesions of BALB/c mice compared to C57BL/6 mice. This indicates that MCs regulate the outcome of leishmaniasis and is dependent on the genetic background of the host (68). Conversely, a recent study showed that MC has no impact on the severity of cutaneous leishmaniasis in mice infected with *L. major*. By using *Kit* mutant mice with different genetic backgrounds, it was shown that MC deficiency did not affect lesion size development after *L. major* infection, suggesting that MCs are not involved in murine cutaneous *Leishmania* infections (30).

### MCs in Visceral Leishmaniasis

Visceral leishmaniasis is a serious public health problem that causes high morbidity and mortality (69). Analysis of Th1, Th2, and Th17 cytokine responses by cultured peripheral blood mononuclear cells from patients who had developed kala-azar caused by *Leishmania donovani*, or who were protected against kala-azar, showed that IL-17 and IL-22 are the cytokines most strongly associated with protection against kala-azar (70). It has been reported that higher numbers of plasma cells, lymphocytes, and Mφ but lower number of MCs are present in the lamina propria of gastrointestinal tract of dogs naturally infected with *L. infantum* compared to non-infected controls, in all gastrointestinal tract segments (31). Thus, MCs may play different roles in visceral leishmaniasis and cutaneous leishmaniasis.

### MCs in Mucocutaneous Leishmaniasis

Mucosal leishmaniasis is a chronic infection that affects the upper respiratory tract and/or the oral mucosa (71). Some patients diagnosed with mucosal leishmaniasis have oral lesions. Scraping cytology examination in patients with oral leishmaniasis presented free Leishman-Donovan bodies or Mφ loaded with Leishman-Donovan bodies, acute and chronic inflammatory cells, histiocytes, multinucleated giant cells, MCs, and plasma cells (72).

### MCs in Ocular Leishmaniasis

Ocular involvement is an unusual presentation of leishmaniasis and commonly limited to the eyelid skin (73). Both C57BL/10 and BALB/c mice are susceptible to leishmaniasis, infected with *L. amazonensis* by intravitreal injection and instillation, respectively. Independent of the infective routes, C57BL/10 mice infected intravitreally presented an intense inflammatory reaction in the epithelium of the eyelids, as well as the presence of many intact MCs in the conjunctiva of the eyes from 30 days postinfection (p.i.). On the other hand, BALB/c mice infected *via* the instillation route presented no lesions but an enhancement of intact MCs in the conjunctival region at 30 days p.i., and a discreet inflammatory infiltrate and degranulated MCs were observed in the conjunctival region at 60 days p.i. (32).

### MC–TLRs Interaction during *Leishmania* Infection

To date, the regulatory effect of MCs on the pathogenesis of leishmaniasis is still unclear. The clearance of *L. major* strongly depends on TLRs (24). TLR9-deficient Mφ had reduced expressions of CD40, IL-12, and TNF-α (74). MCs respond to TLR ligands by secreting cytokines, chemokines, and lipid mediators, and some studies have found that TLR ligands can also cause MC degranulation (75). The HMC-1 stimulated by promastigotes of *L. braziliensis* has a significantly greater release of histamine and IL-4 compared to control cells treated with medium (76). MCs release IL-3 and IL-4 to render Mφ susceptible to *Leishmania* infection *in vitro* (25).

## MCs in *Trypanosoma* spp. Infection

### MCs in *T. cruzi* Infection: Chagasic Megacolon

Megacolon is frequently observed in patients with Chagas disease caused by *T. cruzi* infection, which has been considered a consequence of an inflammatory process, and inflammatory infiltrates are composed of lymphocytes, Mφ, natural killer cells, MCs, and eosinophils. Morphometric analyses in the lamina propria, muscle layer, or myenteric plexus region revealed that the numbers of both tryptase-immunoreactive MCs and eosinophils are significantly increased in patients with megacolon compared to uninfected individuals. MC and eosinophil activation, as well as their physical interaction, were observed by electron microscopy (33). *T. cruzi*-induced injury resulted in intramuscular fibrosis and increased thickness of the colon wall in patients with chagasic megacolon, and there was a greater MC count and more fibrosis in the circular colon musculature of chronic Chagas patients with megacolon compared to Chagas cases without megacolon (34). The density of MCs was significantly higher in the esophagus and large intestine in patients with AIDS plus Chagas disease reactivation compared to chronic chagasic patients without AIDS. This suggests that MCs may play a major role in esophageal and intestinal inflammation during Chagas disease reactivation in HIV-coinfected patients (77). MC-specific proteases (tryptase and chymase) influence the activation of inflammatory cells. Increased numbers of tryptase-immunoreactive MCs were found in the esophagus sections of *T. cruzi*-infected individuals with or without megaesophagus. However, increased numbers of chymase-immunoreactive MCs were only found in the esophagus sections of infected individuals without megaesophagus compared to the control groups. Therefore, patients with megaesophagus had increased levels of tryptase-immunoreactive MCs (14).

One animal study showed that, after infection with the Y strain of *T. cruzi*, there were no significant differences in MC counts in the acute phase in Swiss mice. However, there was a significant increase in the number of MCs in the muscular layer of chronically infected Swiss mice with chagasic megacolon compared to non-infected control mice, accompanied by increased thickness of the colon wall, diffuse muscle cell hypertrophy, and increased collagen deposition (35), which may be associated with MC functions.

### MCs in *T. cruzi* Infection: Chagas Heart Disease

The density of MCs in the myocardium was shown to be significantly higher in the chronic chagasic patients compared to control groups (77). The autopsied chagasic patient group showed higher MC chymase and MC tryptase densities and a higher percentage of collagen in the lingual muscles and myocardium compared to the non-chagasic patient group, and MC chymase level was associated with the intensity of myocardium fibrosis of chronic Chagas disease (37). Infiltrated T cells, Mφ, B cells, and MCs were all observed in the myocardium of patients with Chagas cardiopathy, who died at an early mean age or at older ages. However, the numbers of T-lymphocytes and MCs were significantly higher in the cases who suffered early cardiac death (78).

One animal study showed that *T. cruzi*-infected CBA mice treated with cromolyn (a MC stabilizer) presented much greater parasitemia and IFN-γ levels, higher mortality, myocarditis, and cardiac damage, indicating that MCs control blood and tissue parasitemia, IFN-γ production, cardiac inflammation, and susceptibility to infection, suggesting that MCs are involved in resistance to this infection (36).

### MCs in *T. brucei* Infection

*Trypanosoma brucei* is a protozoan parasite that causes human African trypanosomiasis. Rats initially infected with *T. brucei*, followed by infection with *Trichinella spiralis*, showed that *T. brucei* infection does not significantly alter the number of MCs generated by *T. spiralis* infection, while the intestinal MC numbers in rats infected with only *T. brucei* were similar to those in uninfected rats (38).

## MCs in *T. gondii* Infection

### MCs in Toxoplasmic Encephalitis

Toxoplasmic encephalitis in patients with AIDS is a life-threatening disease, mostly due to the reactivation of *T. gondii* cysts in the brain (79). It has been reported that a patient with meningoencephalitic toxoplasmosis was associated with systemic cutaneous and gastrointestinal mastocytosis, suggesting a possible relationship between MC proliferation and the parasitic infection (39).

### MCs in Ocular *T. gondii* Infection

*Calomys callosus* (Rodentia: Cricetidae) animals were inoculated intraperitoneally or *via* the conjunctiva with tachyzoites of the RH strain of *T. gondii*, resulting in the presence of the parasites and inflammatory cells and a significant increase in the number of MCs. Furthermore, MC activation in the ocular tissues was observed after infection, suggesting that MCs play an important role in the acute inflammatory response against *T. gondii* (40).

### MCs in Oral *T. gondii* Infection

Oral infection is the natural toxoplasmosis route, and the MC population is highly abundant in intestinal mucosa. In MC-deficient mice (W/W^v^) and their control +/+ counterparts orally infected with cysts of the ME49 strain of *T. gondii*, rapid lethality and decreased IFN-γ levels were observed in the serum of infected mice in the absence of MCs. This demonstrated that MCs play a primordial role in resistance to oral infection with *T. gondii*, and MCs are required for survival of mice after oral infection with *T. gondii* (41). RBL-2H3 MCs infected with *T. gondii* type I (RH), II (PTG), or III (CTG or VEG) tachyzoites showed that acute *T. gondii* infection inhibits antigen-mediated MC degranulation, irrespective of the genotype of parasite used, and that tachyzoite attachment but not invasion is necessary for inhibiting degranulation. Ca^2+^ mobilization is a central and well-studied aspect of IgE/FcεRI-mediated signaling in MCs, and *T. gondii* infection has been shown to inhibit MC degranulation by suppressing antigen-mediated Ca^2+^ responses (80).

### MCs in Intraperitoneal *T. gondii* Infection

After intraperitoneal infection with the RH strain of *T. gondii*, the number of non-degranulated MCs was significantly lower than that of degranulated cells in the peritoneal cavity, submandibular and dorsal lymph nodes, and ileum in infected *C. callosus* compared to uninfected animals. After the MC degranulation, a remarkable increase in the influx of neutrophils and Mφ but a decrease in lymphocyte influx toward the peritoneal cavity of the infected animals were observed. MCs were observed interacting with other parasitized cells including Mφ, and extracellular parasites were destroyed during the interaction with MCs exhibiting degranulation. This suggests that MC is an important cell type during the inflammatory response against *T. gondii* (42). The use of MC-deficient *Kit^W^*/*Kit^W-v^* mice demonstrated that the influx of Ly6G^+^ cells toward the peritoneal cavity was significantly reduced compared to control littermates, indicating that MCs are an important chemokine source driving PMN recruitment to the peritoneal cavity during *T. gondii* infection (43). In both wild-type and serglycin-deficient mice, intraperitoneal infection with *T. gondii* resulted in highly increased extracellular levels of glycosaminoglycans, including hyaluronan and chondroitin sulfate A, suggesting that serglycin proteoglycan is dispensable for normal secretion and activity of MC proteases in response to *T. gondii* infection (81). A murine model showed that infection of *T. gondii* increased not only the number of MCs at the site of infection but also a noticeable degree of MC degranulation. Kunming outbred mice were infected intraperitoneally with the RH strain of *T. gondii* and treated by compound 48/80 or disodium cromoglycate (a MC stabilizer). The MC activator aggravated the pathology and increased the parasitic load, accompanied by upregulated mRNA levels of Th1 cytokines (IFN-γ, IL-12p40, or TNF-α) in the livers and spleens of *T. gondii*-infected mice. Conversely, the MC stabilizer improved the pathology and decreased the parasitic load, accompanied by increased mRNA levels of Th2 cytokines (IL-4 and IL-10) in the livers and spleens of mice infected with this parasite. In addition, significantly increased inflammatory foci of neutrophil infiltrates in different tissues occurred as a result of MC degranulation after the parasite infection (44). Thus, the activation or inhibition of MCs is a key factor determining the fate of the infection and associated immunopathology.

### MC–*T. gondii* Interaction *In Vitro*

When *T. gondii* tachyzoites and MCs were incubated together, the tachyzoites adhered to the surface of the MCs, followed by MC degranulation. MC histamine and LTB_4_ release was significantly increased after incubation with the tachyzoites, which resulted in damage to the tachyzoites. MC-treated tachyzoites were found to be incapable of infection and replication in murine peritoneal Mφ. Therefore, LTB_4_ released by MCs and other inflammatory cells may be a key factor in the host defense against *T. gondii* (82). hMCs co-cultured with *T. gondii* RH tachyzoites that were opsonized with IgG directed against the surface antigen 1 exhibited a polarized degranulation toward the invading parasites and resulted in the death of more than 70% of the parasites during the process. On the other hand, non-opsonized *T. gondii* rapidly infected MCs without triggering any detectable degranulation process, and only 20% of the parasites died during the process. hMC treated with a chymase inhibitor did not affect parasite mortality, whereas hMC treated with a tryptase inhibitor significantly decreased the number of dead parasites in contact with degranulated MCs. Thus, IgG-opsonized *T. gondii* resulted in tryptase-dependent parasite death (83). TS-4 strain *T. gondii* infection significantly increased the expression of metalloproteinases (MMP)-2 and MMP-9 in P815 murine mastocytoma cells, and the invading parasites can elicit Erk1/2 phosphorylation, leading to NF-κB activation in the cytoplasm. This pathway for generating MMP-2 and MMP-9 is important in host defense mechanisms against *T. gondii* (84). The mediators of activated MCs play an important role in modulating acute inflammatory pathogenesis and parasite clearance in *T. gondii* infection (85).

## Concluding Remarks

In this review, we have highlighted that MCs influence the outcome and immune response to *Plasmodium* spp., *Leishmania* spp., *Trypanosoma* spp., and *T. gondii* infections, and these protozoan parasites can all trigger MC activation, exhibiting an increase in the number of MCs and the degree of their degranulation, and have fundamentally diverse impacts on protozoan infections in different settings, i.e., protozoan parasite infections can be controlled or may deteriorate through the release of different MC mediators, proteases, and cytokines, etc. In *P. berghei*-infected mice, MCs and MC-derived TNF play protective roles in murine malaria. In *Leishmania* infection, the MC count is positively associated with the disease duration of cutaneous leishmaniasis. During *T. gondii* infection, an increased MC number and greater MC activation are observed in infected animals. Furthermore, MCs are required for mouse survival after oral infection with *T. gondii*. However, MCs can also worsen the outcome of a protozoan infection under certain circumstances. For example, MC degranulation is significantly correlated with parasitemia and disease severity in human malaria; histamine-mediated signaling contributes to malaria pathogenesis. In *T. cruzi* infection, greater MC counts with more fibrosis are found in the colon musculature of chronic Chagas patients with megacolon or the myocardium of patients with Chagas cardiopathy. MC activation by MC stimulators can deteriorate the pathology and increase the parasitic load in acute *T. gondii*-infected mice. Moreover, some studies have shown that MCs have no impact on malaria pathogenesis caused by *P. berghei* ANKA and no effect on lesion size development by *L. major* infection in mouse models. In addition, the numbers of MCs in the intestines of *T. brucei*-infected rats are not significantly different compared to uninfected controls. Mediators from MCs play a key role in inflammation and in the pathogenesis of the protozoan parasitic diseases. Understanding the mechanisms by which MCs regulate pathogenesis during different protozoan parasite infections may potentially lead to the development of a new and unique therapeutic target for protozoan-related diseases. Therefore, further studies that evaluate the clinical importance of MC-protozoan interactions may lead to new therapeutic approaches for these protozoan parasitic diseases.

## Author Contributions

FL conceived and wrote the manuscript, and SH participated in the writing.

## Conflict of Interest Statement

The authors declare that the research was conducted in the absence of any commercial or financial relationships that could be construed as a potential conflict of interest.
